# Anti-beta2-glycoprotein I IgG antibodies are associated with early-onset cryptogenic ischemic stroke

**DOI:** 10.1093/esj/23969873251351207

**Published:** 2026-01-01

**Authors:** Nina Jaakonmäki, Tuukka Helin, Timea Szanto, Marialuisa Zedde, Tomi Sarkanen, Nicolas Martinez-Majander, Juha Sinisalo, Ulla Junttola, Petra Redfors, Bettina von Sarnowski, Ulrike Waje-Andreassen, Pauli Ylikotila, Nilufer Yesilot, Kristina Ryliskiene, Lauri Tulkki, Laura Amaya Pascasio, Radim Licenik, Phillip Ferdinand, Eva Gerdts, Dalius Jatužis, Alessandro Pezzini, Janika Kõrv, Juha Huhtakangas, Ana Catarina Fonseca, Lotta Joutsi-Korhonen, Hugoten Cate, Pekka Jäkälä, Jukka Putaala

**Affiliations:** Neurocenter Neurology, Kuopio University Hospital, Finland and University of Eastern Finland, Kuopio, Finland; Department of Clinical Chemistry, HUS Diagnostic Center, Helsinki University Hospital and University of Helsinki, Helsinki, Finland; Coagulation Disorders Unit, Department of Hematology, Comprehensive Cancer Centre, Helsinki University Hospital, Helsinki, Finland; Neurology Unit, Stroke Unit, Azienda Unità Sanitaria Locale – IRCCS di Reggio Emilia, Italy; Department of Neurology, Tampere University Hospital, Wellbeing Services County of Pirkanmaa, and Faculty of Medicine and Health Technology, Tampere University, Tampere, Pirkanmaa, Finland; Department of Neurology, Helsinki University Hospital, and University of Helsinki, Helsinki, Finland; Cardiology, Helsinki University Hospital and University of Helsinki, Helsinki, Finland; Clinical Neuroscience Research Unit and Department of Neurology, Oulu University Hospital, Oulu, Finland; Department of Neurology, Sahlgrenska University of Hospital, Gothenburg, Sweden; Department of Clinical Neuroscience, Institute of Neuroscience and Physiology, Sahlgrenska Academy at University of Gothenburg, Gothenburg, Sweden; Department of Neurology, University Medicine Greifswald, Greifswald, Germany; Department of Neurology, Haukeland University Hospital, Bergen, Norway; Department of Neurology, Turku University Hospital and University of Turku, Turku, Finland; Istanbul Faculty of Medicine, Department of Neurology, Istanbul University, Istanbul, Turkey; Faculty of Medicine, Institute of Clinical Medicine, Department of Neurology and Neurosurgery, Vilnius University, Vilnius, Lithuania; Department of Neurology, Helsinki University Hospital, and University of Helsinki, Helsinki, Finland; Department of Neurology, Torrecárdenas University Hospital, University of Almería, Spain; Acute Stroke Centre, North West Anglia NHS Foundation Trust, Peterborough, England, UK; Neurosciences, University Hospitals of North Midlands NHS Trust, Stroke-on-Trent, England, UK; Department of Heart Disease, Haukeland University Hospital, Bergen, Norway; Faculty of Medicine, Institute of Clinical Medicine, Department of Neurology and Neurosurgery, Vilnius University, Vilnius, Lithuania; Department of Medicine and Surgery, University of Parma, Parma, Italy; Stroke Care Program, Department of Emergency, Parma University Hospital, Parma, Italy; Department of Neurology and Neurosurgery, University of Tartu, Tartu, Estonia; Clinical Neuroscience Research Unit and Department of Neurology, Oulu University Hospital, Oulu, Finland; Department of Neurology, Hospital de Santa Maria, Institute of Pharmacology and Neurosciences, Faculdade de Medicina, Universidade de Lisboa, Lisboa, Portugal; Department of Clinical Chemistry, HUS Diagnostic Center, Helsinki University Hospital and University of Helsinki, Helsinki, Finland; Department of Internal Medicine, Maastricht University Medical Center and CARIM School for Cardiovascular Diseases, Maastricht, The Netherlands; Neurocenter Neurology, Kuopio University Hospital, Finland and University of Eastern Finland, Kuopio, Finland; Department of Neurology, Helsinki University Hospital, and University of Helsinki, Helsinki, Finland

**Keywords:** Antiphospholipid antibodies, ischemic stroke, young adult

## Abstract

**Background:**

Previously undetected antiphospholipid antibodies (aPLs) potentially provide explanations for early-onset cryptogenic ischemic stroke (CIS). Prior association studies conducted over a decade ago were inconclusive and not focused on patients with CIS.

**Methods:**

SECRETO is a multi-center case-control study enrolling patients aged 18–49 years with imaging-positive acute CIS and 1:1 matched stroke-free controls. Lupus anticoagulant (LA), anticardiolipin (aCL), and anti-beta2-glycoprotein I (aβ2GPI) IgG antibodies were assessed from blood samples taken at two time points (baseline and 12-weeks) from patients and at a single time point from controls. Conditional logistic regression models assessed the association of aPLs, adjusted for age, level of education, and vascular risk factors.

**Results:**

A total of 503 patient-control pairs were analyzed. At either time-point, compared to healthy controls, patients had more frequently positive aβ2GPI (patients 11.9% vs controls 2.0%, *p* < 0.001). There was no significant difference in the presence of positive LA between patients and controls. In the logistic regression model, at either time-point positive aB2GI and aCL were associated with CIS (odds ratio [OR] 11.22, 95% confidence interval [CI] 4.35–28.95 and OR 20.85, 95% CI 204–213.16, respectively). The frequency of patients with positive aβ2GPI or aCL increased from baseline to 12 weeks (*p* < 0.001), whereas frequency of positive LA results decreased (*p* < 0.001).

**Conclusions:**

Positive aβ2GPI and aCL, but not LA, detected either shortly after stroke or after 12 weeks were associated with early-onset CIS. Notably, after the acute phase, frequencies of positive aβ2GPI and aCL increased, whereas LA showed a reverse trend.

## Graphical Abstract

**Graphical abstract:**
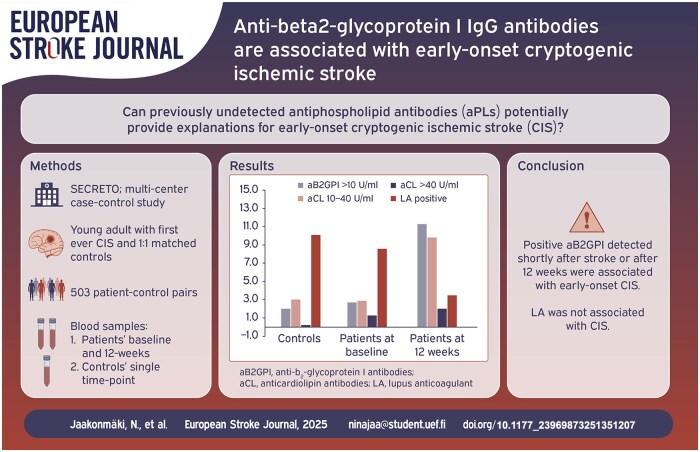

## Introduction

The incidence of ischemic stroke in young adults has constantly increased throughout the past decades and this increase may be largely attributed to the increase of cryptogenic ischemic stroke (CIS) and those without traditional vascular risk factors.^1^ Moreover, patients with early-onset CIS were demonstrated to experience recurrent strokes at a relatively high rate, with a 20-year cumulative risk of 12%.^2^

One group of mechanisms explaining both incident and recurrent ischemic strokes, including CIS, may be disorders of the coagulation system. Some evidence indeed suggests that hypercoagulation would have a stronger association with CIS and play a stronger role in young adults than older adults.^3^ However, there are very scarce data on hypercoagulation for subtypes of early-onset ischemic stroke. Specifically, the association between the most established antiphospholipid antibodies (aPL) – namely lupus anticoagulant (LA), anticardiolipin (aCL), and anti-beta2-glycoprotein I antibodies (aβ2GPI) – and early-onset CIS has been poorly studied.

In established antiphospholipid syndrome – a systemic autoimmune disorder defined by persistently positive aPLs and thrombosis – the incidence of ischemic stroke was reported to be as high as 20%, mostly from large vessel occlusion.^4^ A 2015 systematic review showed an elevated presence of aPLs in early-onset ischemic stroke patients, with high variability of results between the studies.^5^ Notably, many of the studies enrolled only a modest number of participants, varied in laboratory methods and cutoff values, and were performed over a decade ago after which laboratory methods also have evolved. In the seminal RATIO (The Risk of Arterial Thrombosis in Relation to Oral Contraceptives) study, tested at a single time-point, LA increased the risk of ischemic stroke of any etiology in young women over 40-fold, aβ2GPI over twofold, while aCL did not increase the risk of stroke.^6^

We hypothesized that there would be an association with one or several elevated, previously undetected aPL and early-onset CIS and tested this hypothesis in an international multicenter case-control study.

## Methods

The data was based on the Searching for Explanations for Cryptogenic Stroke in the Young: Revealing the Etiology, Triggers, and Outcome (SECRETO; NCT01934725) study, which is a prospective case-control multi-center study, including patients aged between 18 and 49 years with a first ever CIS. For each patient, a sex- and age-matched (±5 years) stroke-free control from the same region was enrolled according to STROBE guidelines.^7^ The study design has been described in detail previously.^8^ Participants were enrolled between October 2013 and November 2022. A written informed consent was obtained from each participant, and the study was approved by the ethics committees of the participating hospitals.

All patients underwent thorough diagnostic workup, and inclusion required diagnosis of CIS, defined according to the ASCO (Atherosclerosis, Small vessel disease, Cardiac source, Other cause) classification system, as either the absence of disease (grade 0), or as grade 2 (causality uncertain) or grade 3 (unlikely a direct cause) pathology.^9^

Prior to inclusion, all patients had to undergo brain magnetic resonance imaging; intracranial and extracranial vascular imaging with either computed tomography angiography or magnetic resonance angiography, routine laboratory tests, 12-lead electrocardiography, prolonged continuous electrocardiography for a minimum of 24 h, and transthoracic and transesophageal echocardiography, according to a prespecified protocol.^10^ Transcranial Doppler (TCD) ultrasound with a bubble screen was performed at selected study centers.^11^

## Investigational blood samples

Investigational blood samples were obtained from study participants for coagulation testing and processed according to the study protocol. We collected blood samples from patients at two time points; at baseline and at 12 weeks, to allow discrimination between changes related to acute stroke or preceding conditions, such as infections, from permanent pathologies and to confirm positive findings. Blood samples were collected from the stroke-free controls at one time point.

Citrated plasma was prepared by a single centrifugation at room temperature at 2000*g* for 10 min. The plasma samples were aliquoted in cryovials, stored locally at study sites for 6–12 months at minimum of −70°C and transferred to Institute for Molecular Medicine Finland (FIMM), University of Helsinki, for storage at −180°C liquid nitrogen gas phase. Courier service used sophisticated systems to maintain stable circumstances during transportation.

All the assays for aPL were assessed centrally at HUS Diagnostic Center (Helsinki University Hospital, Finland). LA was screened with two different tests: one based on activated partial thromboplastin time (APTT) and one on diluted Russell’s viper venom time (dRVVT), using Siemens Healthineers Dade Actin FSL (APTT) and LA1 (RVVT) reagents. Siemens BCS XP coagulation analyzer was used. LA was measured according to CLSI (Clinical and Laboratory Standards Institue) and ISTH (International Society on Thrombosis and Heamostasis) guidelines using both screening and confirmation reagents, and in-house cutoff values.^12–14^ If LA was found borderline or positive, further testing was done, including confirmation, and if needed, mixing tests for both test types (LA2 for dRVVT and Actin FS for APTT).

aCL IgG and aβ2GPI IgG were tested using EliA™ antiphospholipid tests by Phadia™ Laboratory System (Thermo Fisher Scientific). Medical information was gathered from both patients and controls, and the use of oral anticoagulants, antiplatelet medication, low-molecular-weight heparin, or statins at the time of laboratory samples being taken. Since anticoagulation can lead to false positive LA, LA was interpreted as negative, if the study subject was using any oral anticoagulant or therapeutic dose of low-molecular-weight heparin at the time of samples taken.^13^ Cutoffs for positive assay were based on international guidelines and verified in-house.^8^ LA was absent, borderline or present in plasma, aCL of IgG isotype was present in plasma in medium or high titer, suggestive of aPL (i.e. >40 GPL or MPL, or >99th percentile of time) and/or aβ2GPI I IgG isotype was present in plasma in titer >99th percentile. In addition, a borderline finding of positive aCL at low titer (10–40 U/ml) was assessed.

## Comorbidities and lifestyle factors

Comorbidities and lifestyle factors were identified using structured interviews and medical records. Based on generally accepted knowledge, we included the most relevant traditional and nontraditional risk factors associated with early-onset ischemic stroke in our analyses: diabetes mellitus, history of cardiovascular disease, history of venous thrombosis, hypertension, hypercholesterolemia, patent foramen ovale (PFO), cigarette smoking, physical inactivity, heavy alcohol consumption, unhealthy diet, abdominal obesity, psychosocial stress, depression, family history of stroke, and in women, use of estrogen (Table S1).

## Statistical analysis

We performed statistical analyses with IBM SPSS Statistics for Windows, version 27.0 (IBM Corp., Armonk, N.Y., USA) and R (http://www.r-project.org) version 4.0.2. *p*-Values <0.05 were considered significant.

Univariable associations between the predictors and CIS were assessed using statistical testing appropriate for matched case-control studies: McNemar’s test for dichotomized variable, Paired *t*-test to compare normally distributed continuous variables, and Wilcoxon signed rank test for non-normally distributed continuous variables. When patients or controls were analyzed separately, we compared categorical variables with the Chi square or Fisher’s exact test. If a laboratory value was below the lowest cutoff value, the laboratory value was imputed to a numeric value as mid-point between 0 and the lowest cutoff value. Similarly, a laboratory value above the highest cutoff value was imputed to a numeric value as the highest cutoff value +1.

Associations between aPL and CIS were studied using results at different time points: positive results from patients at (a) baseline only, (b) 12 weeks only, (c) both time-points, (d) either time-point, or (d) negative results at both time-points. The results were compared with those of controls taken at one time-point.

Any potential imbalances between patients and controls were addressed using conditional logistic regression analysis for a matched case-control study and produced adjusted odds ratios (OR) with 95% confidence intervals (CI). We adjusted for prespecified confounding factors (diabetes mellitus, hypertension, current tobacco smoking, abdominal obesity, heavy alcohol consumption, physical inactivity, stress, depression, migraine with aura, and in women, use of estrogen).

We further formally tested potential interactions by entering a product variable into the model. In an exploratory analysis, we tested whether there were interactions in the main analysis between aPL and the presence or absence of comorbidities. For this analysis, we used an interaction term in unmatched logistic regression models.

Participants with no blood samples obtained for central analysis were excluded. If a sample was incorrectly labeled or some information about cryovial content was missing, we explored the full electronic sample log maintained by FIMM to handle discrepancies. We imputed missing values for clinical variables with ⩾10 missing values using Multivariate Imputations by Chained Equations R package. Regarding remaining missing values, we reported the number of participants with missing data and excluded them from the multivariable models.

Samples size estimations assumed an alpha of 0.05, a power of 90%. Power estimations and prevalence among control subjects vary with the factor in question and were based on estimates in healthy adults.^15^ A sample of 450 case-control pairs with a prevalence of 15% in patients and 5% in controls (e.g. for any aPL) allows detection of an association with OR of 2.1.

## Results

We identified 546 patients and 546 sex- and age-matched healthy controls. Of these, 43 patient-control pairs were excluded, due to lack of aPL sample analyses (Supplemental Figure S1). Thus, 503 pairs were available for analyses (median age of male and female patients 41.8, IQR 36–46, and 39.2, IQR 30–45, respectively). The proportion of men (54.3%) slightly exceeded that of women. The median delay from stroke to study sample was 8 days (IQR 6 days) and the median NIHSS score on admission was 2 (IQR 0–4). Compared to controls, patients with CIS had a lower level of education, and more frequently abdominal obesity, excessive alcohol consumption, current tobacco smoking, physical inactivity, stress, depression, migraine with aura, and an unhealthy diet (Table 1).

**Table 1.** Clinical characteristics of the included young patients with cryptogenic ischemic stroke and healthy controls, stratified by sex.

Clinical characteristics	All		Men		Women	
	Patients (*n* = 503)	Controls (*n* = 503)	Patients (*n* = 273)	Controls (*n* = 273)	Patients (*n* = 230)	Controls (*n* = 230)
Age, years	40.5 (33.3–45.6)	41.2 (33.4–46.0)	41.8 (35.7–46.2)	41.7 (35.2–46.8)	39.2 (29.9–44.8)	39.5 (29.8–44.9)
Low level of education	273 (54.3)	179 (35.9)*	156 (57.1)	98 (36.3)*	117 (50.9)	81 (35.4)*
Hypertension	173 (34.4)	144 (28.7)	101 (37.0)	91 (33.3)	72 (31.3)	53 (10.6)
Hypercholesterolemia	11 (2.2)	24 (4.8)*	9 (3.3)	20 (3.7)*	2 (0.9)	4 (1.7)
Diabetes mellitus	14 (2.8)	10 (2.0)	9 (3.3)	10 (1.8)	5 (2.2)	0*
Abdominal obesity	297 (59.0)	229 (45.9)*	201 (73.6)	168 (33.7)*	96 (41.7)	61 (12.2)*
Cardiovascular disease	11 (2.2)	5 (1.1)*	8 (2.9)	2 (0.8)	3 (1.5)	3 (1.3)
Excessive alcohol use	72 (14.3)	35 (7.0)*	44 (16.1)	17 (6.3)*	28 (12.2)	18 (7.8)
Current smoking	166 (33.0)	77 (15.4)*	100 (36.6)	41 (15.2)*	66 (28.7)	36 (7.2)*
Physical inactivity	134 (26.6)	106 (21.4)*	68 (24.9)	57 (21.2)	66 (28.7)	49 (21.6)
Depression	156 (31.1)	113 (22.9)*	78 (28.6)*	47 (17.7)	78 (34.1)	66 (29.1)
Psychosocial stress	253 (25.3)*	206 (41.4)	130 (47.6)*	98 (18.0)	123 (53.7)	108 (47.4)
Unhealthy diet	264 (52.5)	186 (37.3)*	154 (56.4)	118 (43.7)*	110 (47.8)	68 (29.8)*
Estrogen use	NA	NA	NA	NA	59 (25.7)*	41 (8.2)
Migraine with aura	216 (42.9)	83 (16.5)*	94 (34.4)	34 (12.5)*	122 (53.0)	49 (21.4)*
History of venous thrombosis	18 (3.6)	4 (0.8)*	7 (2.6)	2 (0.4)	11(2.2)	2 (0.4)*
Family history of stroke	257 (54.4)	218 (44.9)*	134 (53.0)	109 (41.8)*	123 (56.2)	109 (48.7)
Family history of stroke at age < 50 years	35 (7.4)	16 (3.3)*	14 (5.5)	5 (1.9)*	21 (9.6)	11 (4.9)

NA: not applicable.

Data are *n* (%) or median (interquartile range).

^*^ *p* < 0.05.

**Figure 1.**
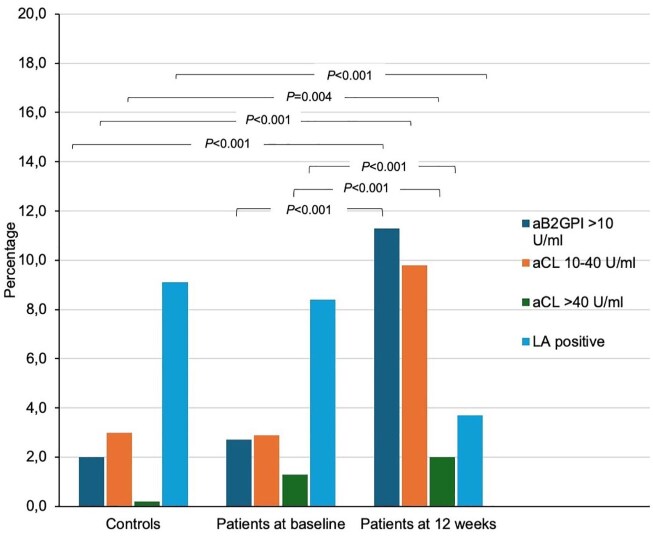
Prevalence of antiphospholipid antibodies in young ischemic stroke patients at two time-points and healthy controls at one time-point. aβ2GPI: anti-β2-glycoprotein I antibodies; aCL: anticardiolipin antibodies; LA: lupus anticoagulant. *p*-Values are for comparison of patients and controls, and for change in patients at baseline and 12 weeks.

Overall, 54 (11.2%) and 70 (15.5%) patients were tested positive for aPL at baseline and 3 months, respectively, with 56 (11.4%) controls being aPL positive. Clinical characteristics of patients with positive aPL results were similar to those with aPL negative results (Table S2). The frequencies of positive aβ2GPI and low-to-high titer aCL increased from baseline to 12 weeks in patients, and on the opposite, the frequency of positive LA decreased (Table 2 and Figure 1). LA positive study subjects anticoagulated the time of the samples taken were regarded as negative due to unreliability of results (baseline controls 0%, patients 6.1%, and 12-weeks 5.3%; Table 2). In further analyses of LA results, positive results in both RVTT and APTT were found in 0% of controls, 1.4% of patients at baseline and 0.6% of patients at 12 weeks (Table S3).

**Table 2.** Comparison of the presence of antiphospholipid antibodies in healthy controls versus baseline and 3-month blood samples of young cryptogenic stroke patients.

aPL (*n* missing, controls/patients baseline/patients 3 months/patients both time-points	Controls (*n* = 503)	Patients at baseline (*n* = 503)	Patients at 12 weeks (*n* = 503)	Patients at both time-points (*n* = 503)	Patients at either time-point (*n* = 503)
LA (7/18/53/60)					
Positive	46 (9.1)	41 (8.4)	16 (3.6)*	9 (2.0)*	48 (10.8)
Borderline	3 (0.6)	0	0	0	0
Anticoagulant^¶^	0	30 (6.1)*	24 (5.3)*	11 (2.2)†	43 (9.6)*
Positive or borderline	49 (9.9)	41 (8.4)	16 (3.6)*	9 (2.0)*	48 (10.8)
aβ2GPI (10/34/63/75)					
Positive (>10 U/ml)	10 (2.0)	13 (2.7)	51 (11.3)*	13 (3.0)	51 (11.9)*
aCL (11/31/64/74)					
Positive (>40 U/ml)	1 (0.2)	6 (1.3)	9 (2.0)†	5 (1.2)	10 (2.3)^†^
Low titer (10–40 U/ml)	15 (3.0)	14 (2.9)	44 (9.8)*	11 (2.6)	47 (10.9)*
aβ2GPI and/or aCL (12/28/57/81)					
Both positive	1 (0.2)	2 (0.4)	4 (0.4)	1 (0.2)	5 (1.2)
Either one positive	10 (2.0)	17 (3.5)	56 (12.4)*	17 (3.4)	56 (13.1)*
aβ2GPI positive and aCL low titer	2 (0.4)	5 (0.5)	30 (6.7)*	3 (0.6)	32 (7.4)*

aPL: antiphospholipid antibody; LA: lupus anticoagulant; aCL: anticardiolipin antibodies; aβ2GPI: anti-β2-glycoprotein I antibodies.

Data are *n* (%). Positive or borderline refers to definite results (without anticoagulation).

^¶^Positive, but result is not reliable due to use of any oral anticoagulant or therapeutic dose of low-molecular-weight heparin at time of samples taken.

^*^ *p* < 0.001 for comparison to controls; ^†^ *p* < 0.05 for comparison to controls.

At either time-point, compared to healthy controls, patients had more frequently positive aβ2GPI (11.9% of patients, 2.0% of controls, *p* < 0.001) and positive aCL (2.3% of patients vs 0.2% of controls, *p* = 0.007). Double-positive aPL results in patients remained scarce (Table 2).

After adjusting for age, level of education and vascular risk factors, positive aβ2GPI and positive aCL were both associated with an increased risk for CIS. Further adjusting for migraine with aura, the risk for CIS was further increased for both aβ2GPI and aCL, but simultaneously widened the confidence interval (Table 3). Multivariable model results considering positive LA regardless of anticoagulation appear in Table S4.

**Table 3.** ORs and 95% CI from conditional logistic regression on the association of antiphospholipid antibodies with cryptogenic ischemic stroke in young adults, stratified by sex. Antiphospholipid antibodies present at either time-point in patients compared to one time-point in controls.

n = 503 patient-control pairs	Model adjusted for age and level of education	Model adjusted for age, level of education, and vascular risk factors*	Model adjusted for age, level of education, vascular risk factors*, and migraine with aura
All			
LA	1.36 (0.80–2.31)	1.13 (0.63–2.03)	1.08 (0.59–2.00)
aβ2GPI	6.32 (2.80–14.20)	7.77 (3.19–18.92)	10.18 (3.88–26.68)
aCL	12.20 (1.49–100.14)	14.43 (1.53–135.65)	20.85 (2.04–213.16)
aβ2GPI and/or aCL	7.25 (3.23–16.25)	8.33 (3.47–19.98)	11.22 (4.35–28.95)
Men			
LA	1.93 (0.97–3.84)	1.68 (0.73–3.89)	1.62 (0.69–3.80)
aβ2GPI	4.42 (1.66–11.77)	5.59 (1.73–18.09)	8.35 (2.33–30.01)
aCL	9.74 (1.11–85.08)	9.31 (0.77–111.20)	10.62 (0.76–147.79)
aβ2GPI and/or aCL	5.57 (2.10–14.76)	6.18 (1.99–19.17)	9.20 (2.66–31.87)
Women			
LA	0.76 (0.32–1.82)	0.75 (0.28–1.99)	0.74 (0.26–2.10)
aβ2GPI	11.88 (2.68–52.65)	14.51 (3.06–68.82)	20.49 (3.93–106.97)
aCL	NA	NA	NA
aβ2GPI and/or aCL	12.03 (2.73–53.08)	14.74 (3.12–69.68)	21.69 (4.16–113.20)

LA: lupus anticoagulant; aCL: anticardiolipin antibodies; aβ2GPI: anti-β2-glycoprotein I antibodies, NA: not applicable.

Due to low frequency of positive results, aCL was not computed for women. Data are odds ratio (95% confidence interval).

^*^Hypertension, diabetes mellitus, hypercholesterolemia, current tobacco smoking, physical inactivity, excessive alcohol use, stress, depression, unhealthy diet, abdominal obesity, and in women, estrogen use. In women, diabetes mellitus and hypercholesterolemia were excluded due to their low frequency.

The interaction analyses suggested that, among individuals with positive aβ2GPI, use of estrogen potentiates the risk for CIS (*p* = 0.043, Table S5). We found no interaction between use of estrogen and aCL or LA, or between current smoking and any of the aPLs (Tables S6–S8). Finally, patients with positive aPL did not have a high-risk PFO more often than aPL negative patients (Table S9).

## Discussion

Our study brings novel insight into the role of aPLs as risk factors for early-onset CIS without known APS. In our study, LA did not increase the risk of CIS, not even when interactions with current smoking and estrogen use were considered. In contrast, aCL and particularly aβ2GPI were stronger risk factors for CIS than previously noted in studies not focusing specifically on CIS. After rigorous adjusting for demographics and traditional and non-traditional risk factors, aβ2GPI increased the likelihood for CIS, especially for women.

Our study results are partly in contradiction to previous studies. The RATIO study found that LA increased the risk of ischemic stroke of any etiology in young women, with an OR of 43.1 (95% CI 12.2–152.0) and in the 2015 systematic review, the frequency of LA in stroke patients was 15.8%.^5,6^ The LA test is a not well-standardized test, that still varies in testing methods, cutoffs, and interpretation from one laboratory to another,^12,14^ which may explain some of the differences between previous studies, and those and our studies. Our study was executed according to current guidelines, and in accordance, we minimized the risk of false positive results by regarding anticoagulated LA positive results as negative (9.6%). Moreover, it should be emphasized that most of the previous association studies on LA have not focused on early-onset CIS.

Compared to several previous studies, aβ2GPI appears to be a stronger probable risk factor for particularly early-onset CIS. The 2015 systematic review indicated a 13.7% frequency of aβ2GPI in ischemic stroke patients of all ages, and the RATIO study found an increased risk of ischemic stroke of any etiology in young women with an OR of 2.3 (95% CI 1.4–3.7).^5,6^ In the 2016 study based on the Danish Stroke Registry, the prevalence of aβ2GPI was not higher in early-onset ischemic stroke patients compared to healthy controls, but the study neither was focused on CIS patients.^16^ However, there is accumulating evidence of the relevance of aβ2GPI in the pathogenesis of thrombosis, as it appears to have both antithrombotic as well as procoagulant effects.^17^ According to current guidelines, our study focused on IgG antibodies, which are more relevant for thrombotic disorders than IgM antibodies that have been used in some of the previous studies.^17^

In accordance with the RATIO study, we did not find any association between aCL and CIS in young women, but the overall presence of aCL remained lower in patients (low titer 9.3% and medium-to-high titer 1.9%) than in the 2015 systematic review (22%).^5,6^ Notably, over half of the studies in the latter analysis used a low aCL cutoff value.

We found aPLs to be a moving target, with a distinct fluctuation of frequencies from baseline to 12 weeks in patients: the frequency of positive LA results decreased and the frequency of low-to-high titers of aCL, as well as frequencies of positive aβ2GPI, increased. One possible explanation could be that lower levels of aCL and aβ2GPI antibodies during the acute phase may be due to their consumption. The acute-phase reactants may transiently suppress aCL and aβ2GPI following an inciting event. Another explanation is that aCL and aβ2GPI antibodies may localize on damaged endothelial cells or platelets at the site of thrombosis, reducing measurable levels in plasma. With such a fluctuation, persistent positive findings from both time-points, therefore fulfilling the APS criteria, remained relatively uncommon. Despite this, positive aβ2GPI in particular appears to be a relevant risk factor for early-onset CIS even without fulfilling the APS criteria. However, we did not take samples from patients beyond 12 weeks and thus cannot demonstrate consistency of the positive results. The fluctuation tendency of aPL results is a distinguished phenomenon.^13^

Our interaction analyses suggested that estrogen use could further increase the likelihood of CIS in aβ2GPI positive patients. Interestingly, high-risk PFO was observed as commonly in aβ2GPI positive patients as aβ2GPI negative patients, suggesting that the presence of aβ2GPI is not specific to PFO-related stroke pathophysiology. Additionally, we found no interaction between any of the aPLs and smoking. In the RATIO study, the risk of ischemic stroke escalated in LA positive women who smoked (OR 87.0, CI 14.5–523.0) or used oral contraceptives (OR 201; CI 22.1–1828.0).^6^

The strengths of our study include a large number of case-control pairs of both sexes, of whom vast data on comorbidities and lifestyle factors were collected and considered in the analyses. Blood samples were taken at two time points and samples were analyzed in one central laboratory, eliminating the risk of variable laboratory methods. Furthermore, we applied solid laboratory methodology and the most recent guidelines to interpret the results.^13^ This study also has limitations. Firstly, there is a risk of selection bias based on the study design. Some patients with the most severe, or morbid stroke could have been left out, yet as only approximately 10% of young stroke patients present with severe symptoms, we can consider our study well representative of young adults.^18^ Secondly, we were unable to predict the trend of the aPLs beyond 12 weeks after stroke. Thirdly, due to the cross-sectional case control-study design, we cannot demonstrate causality.

In stroke patient care, most routine coagulation testing from ischemic stroke patients are performed early on after the stroke and testing at 12 weeks is performed only after a positive acute phase result. However, the results from our study suggest that routine testing for aβ2GPI and aCL at 12 weeks for young CIS patients could be necessary despite the initially negative results from the acute phase, in order to consider appropriately targeted secondary prevention.

## Supplementary Material

sj-docx-1-eso_23969873251351207

## Data Availability

Data will be available upon reasonable request.

## Trial registration

NCT01934725.
